# Value of Intravoxel Incoherent Motion (IVIM) Imaging for Differentiation between Intrahepatic Cholangiocarcinoma and Hepatocellular Carcinoma

**DOI:** 10.1155/2022/1504463

**Published:** 2022-05-10

**Authors:** Jinhua Wang, Zhongxian Yang, Min Luo, Cui Xu, Mu Du, Yubao Liu

**Affiliations:** ^1^Medical Imaging Center, Shenzhen Hospital, Southern Medical University, Shenzhen 518000, China; ^2^The Third School of Clinical Medicine, Southern Medical University, Guangzhou 510060, China

## Abstract

Efficient noninvasive imaging techniques in the differentiation of intrahepatic cholangiocarcinoma (ICC) and hepatocellular carcinoma (HCC) are very important because of their different management and prognosis. Our purpose was to evaluate the difference of parameters extracted from intravoxel incoherent motion (IVIM) diffusion-weighted imaging (DWI) between the two groups and their performance for the differentiation, as well as the significance of perfusion information. IVIM studies (9 *b*-values) in 41 patients with either ICC or HCC were reviewed retrospectively by two observers. Diffusion coefficient (*D*), pseudodiffusion coefficient (*D∗*), perfusion fraction (*f*), ADC, and the mean percentage of parenchymal enhancement (MPPE) at 30 s after contrast-enhancement were calculated and compared between ICC and HCC. The relationship between *D∗*, *f* values, and MPPE was evaluated by Spearman's correlation test. The diagnostic efficacy of all parameters was analyzed by the receiver operating characteristic (ROC) curve. Interobserver and intraobserver agreements were analyzed. The parameters (*D* and ADC) of ICC were distinctly higher than those of HCC; whereas the parameters (*f* and MPPE of arterial phase) were distinctly lower (all false discovery rate [FDR]-corrected *P* < 0.05). The metric *D∗* value of ICC was slightly higher than that of HCC (71.44 vs 69.41) with FDR-corrected *P* > 0.05. Moreover, the value of parameter *D* was significantly lower than that of ADC (FDR-corrected *P* < 0.05). The parameters (*D* and *f* values) extracted from IVIM showed excellent diagnostic efficiency in the identification, and the diagnostic efficiency of *D* value was significantly higher than that of the ADC. There were positive correlations between perfusion-related parameters (*D∗*, *f* values) and MPPE. Interobserver and intraobserver agreements were excellent or perfect in measurements of all parameters. Parameters derived from IVIM were valuable for distinguishing ICC and HCC. Moreover, the *D* value showed better diagnostic efficiency for the differential diagnosis than monoexponential fitting-derived ADC value. Meanwhile, the significant correlation between perfusion-related parameters and MPPE demonstrates that specific IVIM metrics may serve as a noninvasive indicator for the vascular perfusion information of ICC and HCC.

## 1. Introduction

Intrahepatic cholangiocarcinoma (ICC) is characterized by a cholangiocarcinoma occurring proximally to the second-degree bile ducts [1, 2]. ICC and hepatocellular carcinoma (HCC) are the two most common primary intrahepatic malignancies, and prognosis and treatment options differ significantly between them [3, 4]. Therefore, it is important to make an accurate diagnosis and differential diagnosis for ICC and HCC.

As is known, magnetic resonance imaging (MRI) is useful for the detection of HCC and ICC [5, 6]. Typical ICC and HCC emerge with different typical MRI features, such as HCC is characterized by intense arterial uptake and then washout in the venous phase on multiphase imaging techniques, which is distinct from ICC [5–8]. However, some atypical ICC shared many demographic, clinical, and MRI similarities with HCC. It is difficult to distinguish them by using the conventional sequences including contrast enhancement [9, 10].

Recently, the requirement for noninvasive imaging techniques to differentiate liver tumors makes IVIM a hot research topic in the absence of preoperative histopathological confirmation [11–13]. Parameters extracted from IVIM-DWI can reflect both the diffusion and perfusion features of different tissues [14–16]. The diffusion and perfusion features of the tumor displayed by IVIM-DWI depend on the tissue components. ICC is different from HCC because it contains more desmoplastic stroma [14–17]. ICC is composed of large amounts of fibrotic tissue; the fibrotic tissue is a typical feature in distinguishing it from HCC if we can correctly identify it [14–20]. Various reports have described parameters from IVIM or DWI for HCC [21–24]. However, up to now, there were a few reports on the diagnostic value of IVIM parameters and monoexponential fitting-derived ADC for the identification of ICC from ICC as well as comparing their differences [25, 26]. Thus, there is limited available information and no consensus for the assessment of the value of IVIM in differentiation.

Moreover, to our knowledge, no comprehensive data for multiparametric liver MR imaging including IVIM-DWI and the mean percentage of parenchymal enhancement (MPPE) in patients with ICC or HCC exists to date. This study aimed to assess the value of the IVIM-DWI parameters of ICC and HCC as a noninvasive tool for tissue characterization. Besides, we considered the contrast enhancement features and evaluated the correlation between MPPE and perfusion-related parameters from IVIM-DWI of ICC and HCC.

## 2. Experimental Details

### 2.1. Patient Selection

This retrospective study had approval obtained from the institutional review board. Between March 2020 and December 2021 at Shenzhen Hospital of Southern Medical University, China, 41 patients with surgical resection and a definitive diagnosis of either ICC or HCC were involved in this study. The inclusion criteria were as follows: patients with a confirmed diagnosis of ICC or HCC based on histopathologic findings of the fine needle aspiration (FNA) or surgical specimen; patients with an IVIM examination performed within 2 weeks before the FNA or surgery; patients without any therapy including chemotherapy and radiotherapy for the tumor before the IVIM exam. Patients with mixed ICC-HCC or with a measurement of less than 10 mm in size were excluded, and patients who had previous TACE treatment or poor IVIM image quality were excluded. Therefore 41 patients (25 men and 16 women; mean age, 61.24 years; age range, 48–78 years) met the criteria and were involved in the study. According to WHO classification of biliary malignancies, tumors of intrahepatic bile ducts and intrahepatic cholangiocarcinoma were involved in our studies. We excluded combined hepatocellular cholangiocarcinoma. According to the World Health Organization (WHO) classification of tumors, records the histological classification of the HCC group as well differentiated, moderately differentiated, and poorly differentiated.

### 2.2. Protocols of IVIM and Contrast-Enhanced MRI

All images were obtained by a 3.0-T scanner (Signa Excite HD; GE Healthcare, Milwaukee, WI) with a 32-channel phased-array coil. Routine MR imaging was carried on all patients, including three-dimensional liver acquisition and breath-hold fat-suppressed T2-weighted and T1-weighted sequences. Each patient had gone through respiratory training before the scan. The parameters of the axial T2 images were as follows: slice thickness, 6.0 mm; slice gap, 1.0 mm; repetition time(TR)/echo time(TE), 2609/97 ms; matrix, 384 × 384; and field of view(FOV), 38 × 38 cm. The p of axial T1 images were as follows: slice thickness, 6.0 mm; slice gap, 1.0 mm; TR/TE, 4/2 ms; matrix, 260 × 192; and FOV, 36 × 36 cm. Time of routine MR imaging was approximately 7 min. Before the contrast-enhanced MRI examination, IVIM was carried out by a single-shot spin echo-planar imaging (EPI) sequence. Parallel imaging and total 9 *b*-values were used, ranging from 0–1000 s/mm^2^ (0, 20, 40, 80, 100, 200, 400, 800, and 1000). Gradient directions in the lookup table were 3. Axial images of IVIM were obtained with the following parameters: slice thickness, 6.0 mm; slice gap, 1.0 mm; TR, 9000 ms; TE, 52.2 ms; matrix, 128 × 128; number of excitation (NEX) = 2; matrix size, 128 × 128; and FOV, typically 40 × 40 cm (varied for different patients). Acquisition time of IVIM was approximately between 6 min 10 sec to 6 min 40 sec and obtained during respiratory triggered (RT).

A fat-saturated three-dimensional (3D) spoiled gradient-recalled echo (SPGR) sequence was used to complete contrast-enhanced MRI. The contrast agent was GD-DTPA (Mannerist; Bayer healthcare, Berlin, Germany). It was injected by a dose of 0.1 ml/kg and a speed of 2 ml/sec via MEDRAD R power injection system (Bayer Healthcare, Whippany, NJ). The following images were collected after the injection: hepatic arterial phase (30 seconds), portal venous phase (60 seconds), parenchymal phase (180 seconds), and delayed phase (5 minutes).

### 2.3. Image and Related Data Analysis

The biexponential model was used for the quantification of parameters from IVIM. The equation for the calculation was as follows:

(1) $$\begin{array}{c} \frac{S_{b}}{S_{0}}=\left(1-f\right)\cdot \;\exp\left(-b\cdot D\right)+f\cdot \;\exp\left(-b\cdot D\;\right), \end{array}$$

where *S*_0_ is the signal intensity at *b*-value = 0 s/mm^2^, *S*_*b*_ is the corresponding signal intensity at nonzero b-values. Three parameters can be extracted from IVIM: D (pure diffusion coefficient), *D∗* (pseudodiffusion coefficient associated with perfusion), and *f* (microvascular volume fraction related to microcirculation). As *D∗* is obviously higher than *D*, the signal decay from *D∗* can be neglected at a high *b*-value (>200 s/mm^2^). In this context, equation (1) has the following simplification and *D* can be calculated as follows:

(2) $$\begin{array}{c} \frac{S_{b}}{S_{0}}=\exp\left(-b\cdot D\right). \end{array}$$

Then *S*_*b*_ at all *b*-values was fitted to equation (1) with *D* fixed by the Levenberg–Marquardt method. *f* and *D∗* values were subsequently obtained.

The monoexponential model was used for the calculation of the conventional ADC value from the above-mentioned 9 *b*-values by the following equation:

(3) $$\begin{array}{c} \frac{S\left(b\right)}{S\left(0\right)}=\exp\left(-b\cdot A\;DC\right). \end{array}$$

Unenhanced mean signal intensity (MSI) and MSI of arterial phase were measured on a dynamic contrast-enhanced (DCE)-MRI. The mean percentage of parenchymal enhancement (MPPE) of arterial phase was analyzed, MPPE = [MSI of arterial phase－unenhanced MSI)/(unenhanced MSI)] × 100%. MSI was measured on the representative unenhanced T1WI image, so was the average value of the two regions of interest (ROIs) of the lesion.

All images were transferred to a workstation (GE AW4.6) for postprocessing. Multiple ADC (MADC) software was used to yield IVIM parametric maps and quantify corresponding parameters. All measurements were performed by two abdominal radiologists with 6 (reviewer 1-Wang) and 5 (reviewer 2-Yang) years of experiences. Both reviewers were given lectures on the measurements by the radiologist Liu with 15 years of experience in reading abdominal images. The two independent reviewers were blinded to the histopathologic results for each patient and the number of patients. Reviewers placed regions of interest (ROIs) on the slice manifesting the largest level of the lesion. ROIs should encompass the largest solid tissues of lesions, with the exclusion of hemorrhage, necrosis, and cystic areas. For different b-value maps, ROIs were required to replicate to maintain the consistency. For DCE-MRI images and ADC and IVIM parametric maps, the slice and location of ROIs should be as consistent as possible. The value of each parameter should be measured three times and the average value should be recorded.

Interobserver and intraobserver agreements of the measurements for parameters (*D*, *f*, and *D∗*) of IVIM, ADC value, and MPPE were analyzed. To calculate the interobserver and intraobserver agreement, observer 1 made two sets of size measurements, which should be separated by at least four weeks. The interobserver agreements were performed with the two observers' measurements. The data analysis and statistical calculations used data from the first set data of observer 1.

### 2.4. Statistical Analysis

All analyses were performed by the SPSS version 19.0 software (SPSS, Chicago, IL) and MedCalc version 11.4 (MedCalc, Mariakerke, Belgium) software. In measurements (expressed by mean ± standard) deviation of parameters derived from IVIM, ADC value, and MPPE, the intraclass correlation coefficient (ICC) was adopted to analyze the interobserver and intraobserver agreements. According to the value of ICC, there were different levels of agreement to reflect the differences in reliability: excellent, 0.91–1.00; very good, 0.81–0.90; good, 0.71–0.80; moderate, 0.5–0.70; and poor, <0.5. The observer measurements were evaluated by Bland–Altman analysis. The comparisons of two groups with the same sample was performed by Mann–Whitney *U* test. The correlation between IVIM-derived perfusion parameters (*D∗*, *f*) and MPPE was evaluated by Spearman's rank analysis. Two-sided *P* values of less than 0.05 were considered statistically significant. Receiver operating characteristic (ROC) curve analysis was used to calculate the area under the curve (AUC), sensitivity, specificity, and cut-off points of parameters extracted from IVIM and ADC value, respectively. The comparison of ROC curves was assessed by DeLong test. To control for multiple comparisons, a significant threshold of *P* < 0.05 after false discovery rate (FDR) correction (Benjamini–Hochberg procedure) was used.

## 3. Results

### 3.1. Demographic Data

According to the inclusion criteria, forty-one patients were enrolled in the blinded imaging review: 25 men (mean age, 58.21 ± 12.36 years; age range, 48–78 years) and 16 women (mean age, 64.24 ± 10.17 years; age range, 50–77 years). Among all the cases, 23 were histopathologically diagnosed with ICC (13 men and 10 women; mean age, 62.18 ± 13.34 years; age range, 49–77 years), and 18 were histopathologically diagnosed with HCC (12 men and 6 women; mean age, 65.33 ± 13.12 years; age range, 48–78 years). There was no case with a combined ICC and HCC. Among the HCC cases, the final pathological results were as follows: well differentiated (*n* = 4), moderately differentiated (*n* = 6), and poorly differentiated (*n* = 8).

### 3.2. Data Analysis of IVIM

**Figure 1:**
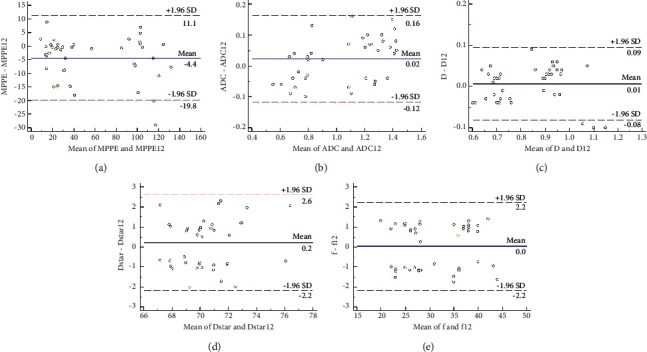
Bland-Altman plots of (a) MPPE, (b) ADC value, (c) *D* value, (d) *D*^∗^ value, and (e) *f* value. The blue and red color indicate the mean difference and limits of agreement, respectively.

Interobserver and intraobserver agreements were excellent or perfect in the measurements of the parameters. Interobserver agreements between observer 1's and observer 2's measurements were excellent for the values of *D*, *f*, *D∗*, ADC, and MPPE. The interobserver ICCs and 95% CIs were: 0.970 (0.943, 0.984), 0.993 (0.986, 0.996), 0.921 (0.853, 0.958), 0.986 (0.973, 0.992), and 0.989 (0.979, 0.994), respectively. The intraobserver ICCs and 95% CIs of the values of *D*, *f*, *D∗*, ADC, and MPPE were 0.977 (0.956, 0.988), 0.993 (0.988, 0.997), 0.912 (0.835, 0.953), 0.984 (0.969, 0.991), and 0.991 (0.983, 0.995), respectively. Bland–Altman plots for the measurements of values of *D*, *f*, *D∗*, ADC, and MPPE showed small absolute intraobserver and interobserver variability (Figure 1).

**Figure 2:**
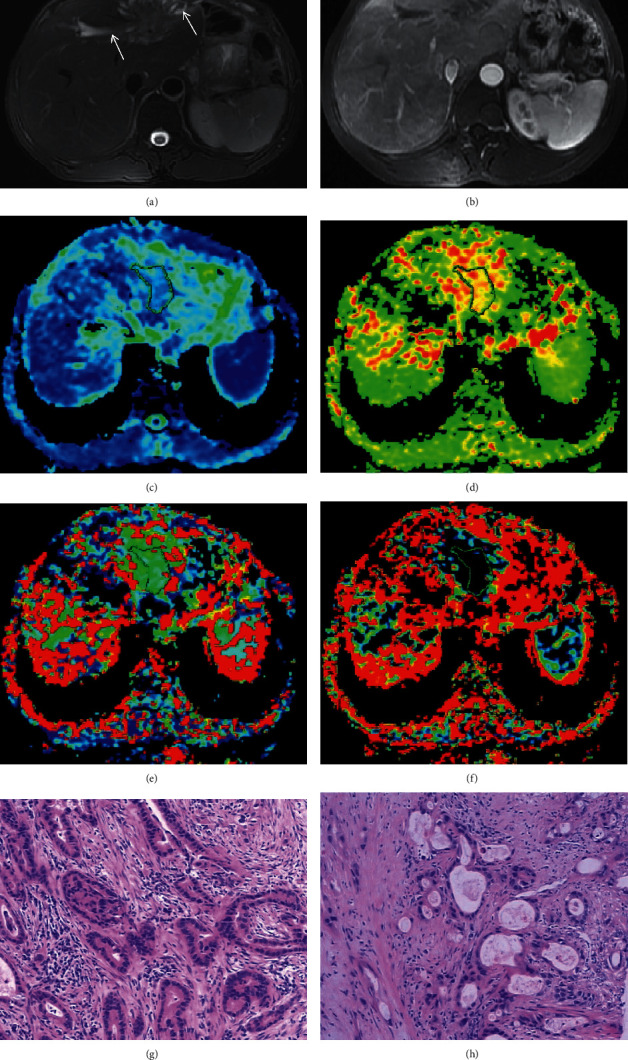
Images of a 56-year-old man, with a histopathological diagnosis of ICC. (a) A fat-suppressed T2-weighted image. The lesion presents with a slightly high signal intensity and the dilation of a peripheral intrahepatic bile duct (white arrow). (b) A contrast-enhanced image of arterial phase. The lesion presents with an inhomogeneous enhancement along with the biliary obstruction. C–F: parametric maps (ADC, *D D*^∗^, and *f* respectively). The lesion presents with different color from the surrounding normal tissue. G-H: HE staining (^∗^100) images show tissues of ICC with dilated bile ducts and rich fibrous stroma.

**Figure 3:**
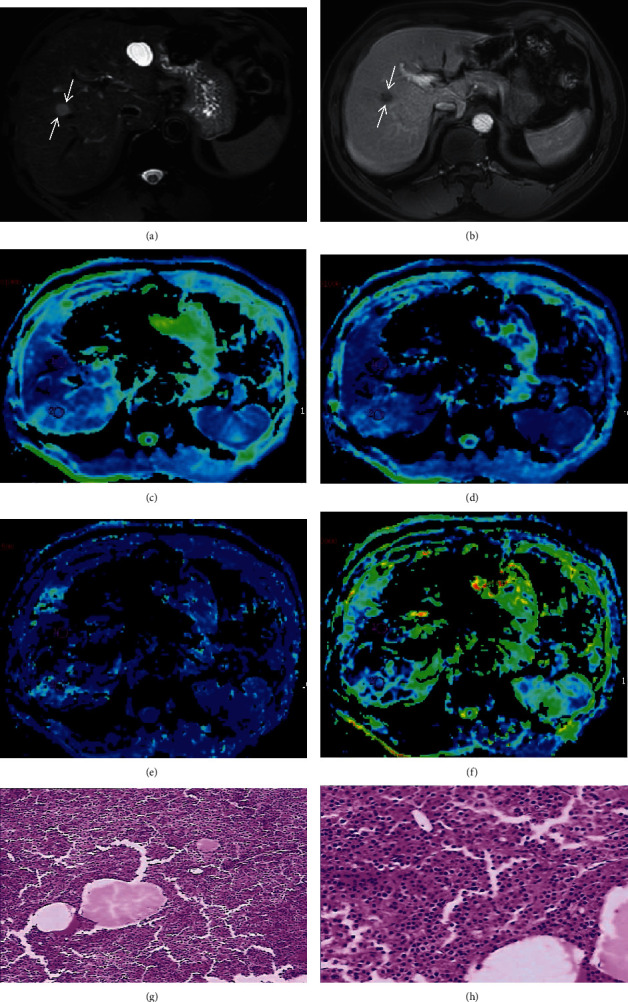
Images of a 47-year-old man, with a histopathological diagnosis of HCC. (a) A fat-suppressed T2-weighted image. The lesion presents with a slightly high signal intensity and a clear boundary (white arrow). (b) A contrast-enhanced image of arterial phase. The lesion presents with an inhomogeneous enhancement. C–F: parametric maps (ADC, *D D*^∗^, and *f*, respectively). The lesion presents with a different color from the surrounding normal tissue. G-H: HE staining (^∗^100) images showed tissues of HCC with high tumor cell density.

Values of parameters (*D*, *f*, and *D∗*) from IVIM biexponential fitting, ADC from monoexponential fitting, and MPPE of ICC and HCC were listed in Table 1 from observer 1. Figures 2–3 show representative HCC and ICC, respectively. *D* and ADC values of ICC (*D*, 0.96 ± 0.06, ADC, 1.31 ± 0.10, 10^−3^ mm^2^/sec) were both higher than those of HCC (*D*, 0.69 ± 0.05, ADC, 0.77 ± 0.15, 10^−3^ mm^2^/sec). Mann–Whitney *U* test demonstrated that *D* and ADC values of ICC were significantly different from HCC (*Z* = −5.44, FDR-corrected *P* = 0.03 for *D*, and *Z* = −5.44, FDR-corrected *P* = 0.03 for ADC). For the comparison of the difference between *D* and ADC value, the *D* value was lower than the ADC value (0.96 vs 1.31 for ICC, FDR-corrected *P* = 0.04; and 0.69 vs 0.77 for HCC, FDR-corrected *P* = 0.04). The *f* value of ICC was obviously lower than that of HCC (25.56 vs 38.29) and the difference were statistically significant (*Z* = −5.44, FDR-corrected *P* = 0.02). *D*^∗^ value of ICC was slightly higher than that of HCC (71.44 vs 69.41), but no significant difference was found between ICC and HCC (*Z* = −2.93, FDR-corrected *P* = 0.95). The MPPE of arterial phase (30 s) of ICC was distinctly lower than that of HCC (21.72 vs 98.28). A significant difference was found in MPPE value between ICC and HCC (*Z* = −5.44, FDR-corrected *P* = 0.01). Spearman's correlation coefficients between perfusion-related parameters (*D* and *f* values) and MPPE are shown in Table 2. There was a good relationship between *D* value and MPPE (*r* = −0.368, *P* < 0.05), as well as the *f* value (*r* = 0.725, *P* < 0.001).

**Figure 4:**
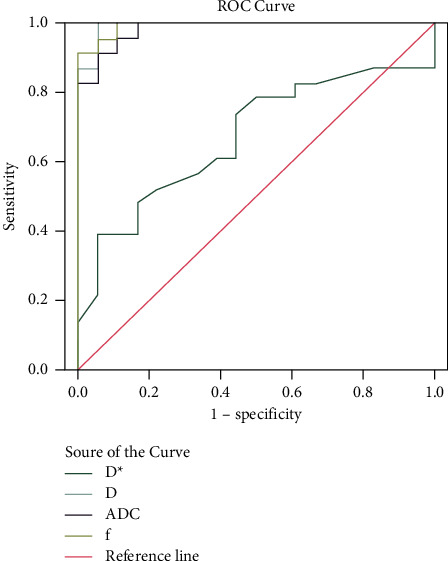
ROC curves of the IVIM-derived parameters and the conventional DWI-derived ADC values for identifying ICC from HCC. AUCs for *D*^∗^, *D*, ADC, and *f* were 0.669, 0.989, 0.960, and 0.973, respectively.

ROC curves obtained for differentiating ICC from HCC are shown in Figure 4. IVIM-derived *D* and *f* values had larger AUCs of 0.989 and 0.973, respectively, than the ADC value with the AUC of 0.960. For the differentiation of ICC and HCC, the comparisons of the ROC curves of *D*, *f*, and ADC values revealed that the *D* value had better diagnostic performance than the ADC value (*Z* = 2.029, FDR-corrected *P* < 0.05), while the diagnostic performance of *f* value was similar to that of the ADC value (*Z* = 0.909, FDR-corrected *P* > 0.05).The *D∗* had lower diagnostic efficacy than the ADC value(AUC: 0.669 vs 0.960, *Z* = 2.909, FDR-corrected *P* < 0.05). Results of the ROC analysis are listed in Table 3.

## 4. Discussion

Traditional DWI and IVIM-DWI are both functional and noninvasive imaging techniques [14–16, 27]. Monoexponential fitting-derived ADC value of traditional DWI comprehensively represents the water molecular diffusion and capillary perfusion, resulting in the lack of reflection of pure water molecular diffusion in tissues [27–29]. Compared with it, IVIM-DWI can extract quantitative parameters (*D*, *D∗*, and *f*) by a biexponential model, which not only reflects the diffusion of water molecules and perfusion of microcirculation in tissues simultaneously but also distinguishes the diffusion information from the perfusion [15, 16, 28, 29]. Some parameters were reported to have significant differences by various studies on hepatic nodules [29–31], but few reports are available on the performance of IVIM for identifying ICC from HCC and the results are inconsistent for different parameters [25, 26]. Our findings of this study may provide useful information for not only the statistical differences of the ADC value and IVIM-derived parameters between ICC and HCC but also the diagnostic performance of them.

Choi et al. [26] carried out a research on IVIM-DWI images of 161 liver nodule cases. They found that HCC had a distinctly lower *D* value than ICC, while its *f* value was higher. The ADC value showed no difference among malignant lesions with different histopathological diagnoses. The *D* value presented the largest AUC compared to other parameters, and the *f* value had a significant positive correlation with the enhancement fraction. However, the study by Wei et al. [32] had different findings: the *D* value can be as helpful as the ADC value for the differentiation of ICC from HCC; the diagnostic efficiency of the *D* value was higher than that of the ADC value, while the *D*^*∗*^ and *f* values presented poor differential diagnostic ability. In addition, Peng et al. [25] reported similar results, but they found that the *D*^*∗*^ value displayed the largest AUC.

This study showed that the *D* and ADC values in the HCC group were both significantly lower than those in the ICC group. Our results are in accordance with previous studies [25, 26, 32]. The possible reason for these results may be the histologic differences of ICC and HCC. HCC is more common than ICC in the appearance of a higher tumor cell density and smaller number of intercellular stroma [33, 34]. Thus, the water molecular diffusion in HCC is more likely to be constrained, resulting in an obvious decrease in diffusion-related parameters (ADC and *D* values), which is reflected by significant decrease in ADC and *D* values. On the other hand, IVIM differs from traditional DWI such that it can distinguish simple water molecular diffusion from microstructural changes within tissues, so microstructural changes for different tissues can be more sensitively and accurately reflected by the IVIM-derived *D* value than traditionally DWI-derived ADC value [27, 28]. According to the theory, another result of our study can be well explained: the *D* value not only decreased lower than ADC within ICC and HCC, but also showed better diagnostic performance for differentiating ICC from HCC in the ROC curve analysis. This result also further confirmed the value of parameter *D* in the differential diagnosis of the two lesions.

As to the perfusion-related parameters (*D*^∗^ and *f* values), the *f* value of ICC was significantly lower than that of HCC; whereas the *D*^∗^ value was slightly higher with no significant difference. Meanwhile, the *f* value showed a higher diagnostic efficiency than the *D*^∗^ value in the ROC curve analysis. The *f* value is the ratio of diffusion associated with microcirculatory perfusion to total diffusion [27, 28]. While the microvessel density of ICC is reported to be significantly lower than that of HCC [35, 36], thereby leading to distinct vascular patterns and a relatively poor blood supply, which is reflected in their imaging characteristics. This may be the explanation for the significant differences of parameters *f* in our study between HCC and ICC. However, our results are inconsistent with previous studies [25, 26, 32]. And the prime reasons may be as follows: (1) The different sample composition and the differentiated degree of HCC in this study. As is reported by Woo et al. [37], when there is a high percentage of well differentiated HCC with a poor blood supply, this may affect the appearance of perfusion-related parameters of the total HCC group. Such a sample composition may result in negative findings in previous studies. (2) Different from the metric *f*, the metric *D*^*∗*^ reflects the diffusion movement information of microcirculation perfusion in the capillary network of the tissue. So, the appearance of *D*^*∗*^ value is susceptible to multiple factors that affect the microcirculation perfusion [38]. It may cause difficulty to accurately assess the *D*^*∗*^ value and thus lead to an insignificant difference and poor diagnostic performance between ICC and HCC. An increased sample size in the further study can possibly solve the problems.

Moreover, a novel finding of this study was that the MPPE of the arterial phase (30 s) of ICC was obviously lower than that of HCC and had a good relationship with the parameters (*D∗* and *f* values) extracted from IVIM. Our results provide an important quantitative supplement to conclusions of current available studies [25, 26]. This result suggests that *D∗* and *f* values extracted from IVIM can be used as an indication of the perfusion of ICC and HCC, which is useful for the diagnosis and differential diagnosis. Furthermore, through the assessment of the perfusion, IVIM-DWI can provide a reliable noninvasive way to evaluate the effect of chemotherapy for either ICC or HCC, which is the main content of our future research.

As is well-known, a biexponential IVIM-DWI model requires at least four different *b*-values [39–42]. Since the application of IVIM-DWI, there have been considerable studies on the selection of b-values [39–42]. It is reported that a large number and wide distribution of b-values increase the reliability and ability of IVIM to reflect pure water molecular diffusion but prolong the image acquisition time [40–42]. Most studies tend to choose approximately 8–14 *b*-values [25, 26, 40–42]. However, it is not practical for clinical application to use a larger number of *b*-values, because the higher number leads to a longer time of image acquisition and may also lead to patient movement. Therefore, the optimized quantity and distribution of *b*-values are very important to both clinical practice and accurate IVIM-DWI parameter measurement. Referring to the considerations and previous literature, 9 *b*-values were used in this study, of which 5 were lower values. In future research, how to make the optimal selection of *b*-values would be further studied.

This study has the following limitations. First, either ICC or HCC, the sample size of those patients was limited. Thus, the correlation between IVIM-derived parameters and the MPPE, histologic characteristics were evaluated in only a subgroup of the whole study population. Second, this retrospective study was carried out in a single center, resulting in only a random choice of ICC according to the available abilities in our center. Meanwhile, there was no prospective selection of this tumor entity. Therefore, this study had inevitable fluctuating patients with large differences in tumor size and location. Third, there was an absence of presentation of biochemical data in this study. And thus, it failed to evaluate the correlation between biochemical indicators and quantitative IVIM parameters to investigate the prognostic value. Fourth, perfusion-related parameters from IVIM, especially the *f* value, are sensitive to the relative T2 differences, and even might be overestimated because of its dependency on TE. Therefore, T2 correction may help the correction of TE dependence, and thus decrease the variability of perfusion-related parameters. However, like previous studies on IVIM for identifying ICC from HCC [25, 26], this study also did not take T2 correction into account, which would be a constructive direction of our further research. Lastly, as no consensus was formed on the quantity and distribution of b-values, this study made the selection of *b*-values mainly according to the demand of clinical applications and previous literature. It is important to keep the balance between the precision of *b*-values and accuracy of parameter measurements, so we should further optimize the quantity and distribution of *b*-values in future research.

## 5. Conclusions

All IVIM-derived parameters can distinguish ICC from HCC, so they are valuable for the diagnosis and differential diagnosis of ICC. Moreover, the diagnostic efficiency of the *D* value is better than the ADC value as well as other parameters. Furthermore, in addition to significant differences of perfusion-related *f* and *D∗* values in ICC and HCC, the correlation between them and MPPE also further indicates that IVIM-DWI may serve as a noninvasive approach to assess the perfusion of HCC and ICC.

## Figures and Tables

**Table 1:** Mean values of IVIM-derived parameters (*D*, *f*, and *D*^∗^) and the conventional diffusion-weighted imaging parameter (ADC), and comparison of them between ICC and HCC groups.

	ICC (*N* = 23)	HCC (*N* = 18)	*P* (FDR-corrected)
*D* (×10^−3^ mm^2^/s)	0.96 ± 0.06	0.69 ± 0.05	0.03
*f* (%)	25.56 ± 2.66	38.29 ± 2.83	0.02
*D∗* (×10^−3^ mm^2^/s)	71.44 ± 2.38	69.41 ± 1.41	0.95
ADC (×10^−3^ mm^2^/s)	1.31 ± 0.10	0.77 ± 0.15	0.03
MPPE	21.72±7.70	98.28 ±21.32	0.01

**Table 2:** Spearman's correlation test between the perfusion parameters extracted from IVIM and MPPE.

	Spearman's correlation coefficients	*P* value
*D* ^∗^	0.623	<0.001
*f*	0.771	<0.001

**Table 3:** Diagnostic values of parameters extracted from IVIM and ADC from the conventional DWI in differentiating ICC from HCC.

Parameters	AUC (95% CI)	Cut-off value	Sensitivity (%)	Specificity (%)
ADC	0.960 (0.861–1.000)	1.25	96.13	54.32
*D*	0.989 (0.917–1.000)	0.77	97.41	82.60
*D∗*	0.669 (0.508–0.734)	28.34	73.40	53.20
*f*	0.973 (0.870–1.000)	0.26	87.24	80.09

## Data Availability

The datasets used and/or analyzed during the the study can be available on reasonable request.
